# Role of substance use in HIV care cascade outcomes among people who inject drugs in Russia

**DOI:** 10.1186/s13722-017-0098-5

**Published:** 2017-12-04

**Authors:** Bulat Idrisov, Karsten Lunze, Debbie M. Cheng, Elena Blokhina, Natalia Gnatienko, Emily Quinn, Carly Bridden, Alexander Y. Walley, Kendall J. Bryant, Dmitry Lioznov, Evgeny Krupitsky, Jeffrey H. Samet

**Affiliations:** 1 Clinical Addiction Research and Education Unit, Section of General Internal Medicine, Department of Medicine, Boston University School of Medicine/Boston Medical Center, 801 Massachusetts Avenue, 2nd Floor, Boston, MA 02118 USA; 2 Clinical Addiction Research and Education Unit, Section of General Internal Medicine, Department of Medicine, Boston Medical Center, 801 Massachusetts Avenue, 2nd Floor, Boston, MA 02118 USA; 3 Department of Biostatistics, Boston University School of Public Health, 801 Massachusetts Avenue, 3rd Floor, Boston, MA 02118 USA; 4 First St. Petersburg Pavlov State Medical University, Lev Tolstoy St. 6/8, St. Petersburg, Russian Federation 197022; 5 Data Coordinating Center, Boston University School of Public Health, 85 E Newton St M921, Boston, MA 02118 USA; 6 HIV/AIDS Research, National Institute on Alcohol Abuse and Alcoholism, National Institute of Health, 5365 Fishers Lane, Bethesda, MD 20892 USA; 7 Pasteur Research Institute of Epidemiology and Microbiology, Mira St. 14, St. Petersburg, Russian Federation 197101; 8 St. Petersburg Bekhterev Research Psychoneurological Institute, Bekhtereva St., 3, St. Petersburg, Russian Federation 192019; 9 Department of Community Health Sciences, Boston University School of Public Health, 801 Massachusetts Avenue, Boston, MA 02118 USA; 10 Department of Infectious Diseases, Bashkir State Medical University, 3 Lenina St., Ufa, Bashkortostan Republic Russian Federation 450000

**Keywords:** HIV care cascade, Russia, Injection drug use, Unhealthy alcohol use, Opioid craving, Linkage to care, ART, Suppressed viral load

## Abstract

**Background:**

Engaging people who drink alcohol or inject drugs in HIV care can be challenging, particularly in Eastern Europe. Healthcare facilities in Russia are organized by specialty; therefore linking patients from addiction care to HIV hospitals has been difficult. The HIV care cascade outlines stages of HIV care (e.g., linkage to care, prescribed antiretroviral therapy [ART], and achieving HIV viral suppression). We hypothesized that unhealthy alcohol use, injection drug use, and opioid craving are associated with unfavorable HIV care cascade outcomes.

**Methods:**

We analyzed data from a cohort (n = 249) of HIV-positive Russians who have been in addiction hospital treatment in the past year and had a lifetime history of injection drug use (IDU). We evaluated the association between unhealthy alcohol use (AUDIT score > 7 [both hazardous drinking and dependence]), past-month injection drug use (IDU), and opioid craving (visual analogue scale from 1 to 100) with HIV care cascade outcomes. The primary outcome was linkage to HIV care within 12 months. Other outcomes were prescription of ART (secondary) and achievement of undetectable HIV viral load (HVL < 500 copies/mL) within 12 months (exploratory); the latter was analyzed on a subset in which HVL was measured (n = 48). We assessed outcomes via medical record review (linkage, ART) and serum tests (HVL). To examine the primary outcome, we used multiple logistic regression models controlling for potential confounders.

**Results:**

Among 249 study participants, unhealthy alcohol use (n = 148 [59%]) and past-month IDU (n = 130 [52%]) were common. The mean opioid craving score was 49 (SD: 38). We were unable to detect significant associations between the independent variables (i.e., unhealthy alcohol use, IDU and opioid craving) and any HIV care cascade outcomes in unadjusted and adjusted analyses.

**Conclusion:**

In this cohort of HIV-positive Russians with a history of IDU, individual substance use factors were not significantly associated with achieving HIV care cascade milestones (i.e., linkage to HIV care; prescription for ART; or suppressed viral load). Given no detection of an association of cascade outcomes with recent unhealthy use of alcohol or injection drugs in this cohort, examining systemic factors to understand determinants of HIV care engagement for people with drug use would be important.

## Background

HIV elimination is a major health target in the United Nations’ (UN) global Sustainable Development Goals, which call for additional resources to effectively address the expanded scope of the HIV epidemic by 2030 [1, 2]. Given that it is challenging to diagnose, link to care, retain, and achieve viral suppression among people with substance use, examining the association of substance use with effective engagement in HIV care is of great interest [3–5].

The HIV care cascade is a framework of consecutive stages of HIV care (i.e., diagnosed, linked to care, retained in care, prescribed ART, and achieved viral suppression) [6, 7]. The HIV care cascade framework is useful for identifying gaps and areas to target HIV interventions [6]. It has been shown that for some HIV-positive individuals, substance use is associated with poor HIV outcomes, even when care is provided free of charge [8–11]. For example, people with unhealthy alcohol or opioid use frequently have reduced adherence to ART medications [12, 13]. As such, people with unhealthy alcohol use and people who inject drugs (PWID) face greater barriers in the path to optimal HIV care and have more rapid HIV disease progression [12, 13]. Understanding the factors that contribute to better HIV care cascade outcomes in a cohort of people who use substances could help to inform strategies to achieve the ambitious UN objectives addressing HIV infection.

Achieving optimal HIV care cascade outcomes is particularly challenging in Eastern Europe. As healthcare services in Russia are organized by specialty [14], linking patients from addiction hospitals to HIV facilities can be a challenging transition [15]. This is mainly important since in the region, the overlapping prevalence of alcohol use, injection opioid use, and HIV infection is very high [16, 17]. The HIV epidemic in Russia has been driven largely by injection drug use, predominantly opioids [18, 19]. In 2015, 17–29% of HIV-positive Russians were estimated to be receiving ART, lower than the global 2015 coverage estimate of 40% and the coverage in the USA and France (70% and 63%, respectively) [20, 21]. The combination of high rates of new HIV infections and low ART coverage contributed to 27,564 HIV-related deaths officially reported in Russia in 2015 [21]. Government statistics put the number of HIV-positive people in Russia over one million [21]. Among those newly diagnosed with HIV in 2015, almost 54% of individuals were infected via injection drug use [21]. People with HIV and substance use comorbidity are a vulnerable population, as their engagement in specialty care remains low [22].

In Russia, healthcare, including addiction and ART, is provided free of charge at governmental facilities, such as addiction (i.e., narcology) or HIV clinics [15]. Opioid agonist therapy with methadone or buprenorphine is not available in Russia [22]. Naltrexone is available for treatment of opioid and alcohol use disorder, but rarely administered due to its cost [23]. The standard of care at Russian inpatient addiction hospitals consists of diagnostic procedures, detoxification for 10–14 days and rehabilitation for an additional 30 days for selected patients. In the first week of hospitalization, patients are detoxified with possible use of tramadol, non-opiate analgesics, clonidine and benzodiazepines [15, 24]. Patients receive drug counselling and treatment for comorbid psychiatric conditions within addiction hospitals, but integration to other treatment modalities such as HIV care is very limited.

The Russian HIV epidemic is a major public health challenge intertwined with substance use, creating a need to better understand barriers to HIV treatment among populations with substance use. Substance use has not been a major focus of previous analyses of the HIV care cascade in Russia. In order to understand whether unhealthy alcohol use, injection drug use (IDU), and opioid craving are associated with HIV care cascade outcomes, we conducted a secondary analysis of prospectively collected observational data about HIV-positive Russians who have been in addiction hospital treatment in the past year and had a lifetime history of IDU. We hypothesized that unhealthy alcohol use, IDU, and opioid craving are associated with unfavorable HIV care cascade outcomes, specifically linkage to HIV care, prescribed ART, and viral load suppression.

## Methods

### Datasets

We conducted a secondary data analysis based on participants from the LINC (Linking Infectious and Narcology Care) study, a randomized controlled trial (RCT) conducted in St. Petersburg, Russia, to assess the effectiveness of a behavioral and structural intervention designed to support and motivate HIV-positive PWID to engage in HIV medical care and ultimately improve their HIV outcomes [25]. LINC participants (n = 349) were recruited from inpatient wards at the City Addiction Hospital in St. Petersburg, Russia between July 2012 and May 2014. Lifetime history of IDU and documented HIV infection were entry criteria. Other inclusion requirements were: (1) aged 18–70 years; (2) hospitalized at the addiction hospital; (3) agree to CD4 cell count testing; (4) having a phone; (5) sharing 2 contacts to assist with follow-up; and (5) residing at a stable address within 100 kilometers of St. Petersburg. Participants were excluded from the study for the following: (1) currently receiving ART; (2) not fluent in Russian; or (3) cognitive impairment precluding informed consent.

The LINC study did not measure HIV viral load (HVL). However, a subset of LINC participants (n = 48) were co-enrolled in another study (Russia ARCH [Alcohol Research Collaboration on HIV/AIDS]) in which the outcome HVL was available. Russia ARCH is an observational cohort of HIV-positive people examining alcohol use and HIV outcomes [26]. Russia ARCH participants were recruited between November 2012 and June 2015 from clinical HIV and addiction sites, non-clinical sites, and via snowball recruitment in St. Petersburg, Russia. Study inclusion criteria were: (1) documented HIV infection; (2) ART-naive at baseline; (3) aged 18–70 years; (4) stable address within 100 km of St. Petersburg; (5) having a phone and; (6) sharing 2 contacts to assist with follow-up. Exclusion criteria were the same as for LINC.

All study participants provided written informed consent and both studies were approved by Institutional Review Boards of Boston University Medical Campus and First St. Petersburg Pavlov State Medical University. Co-enrolled participants provided consent to link their data from the two studies.

### Variable selection

#### Outcomes

The primary outcome of interest was linkage to HIV care. The linkage to care variable was a dichotomous outcome defined as at least one HIV physician appointment within 12 months of study enrollment as all patients were not on ART; this information was obtained from the participants’ medical records [25]. Such an appointment would be made initially at one of two St. Petersburg hospitals serving HIV-positive patients.

The secondary outcomes were prescription of ART (yes or no) and achievement of viral control (exploratory). We defined prescription of ART as being prescribed ART within 12 months following the baseline assessment. This variable was obtained via medical records. We considered achievement of viral control, any HVL < 500 copies/mL within a year of study enrollment. HIV viral load data was obtained via serum tests. This variable was only assessed among LINC participants who were co-enrolled in Russia ARCH.

#### Main independent variables

We assessed 3 key substance use variables at 6 months post-baseline: unhealthy alcohol use, past-month IDU, and opioid craving. Alcohol use was measured via the Alcohol Use Disorder Identification Test (AUDIT, score ranging from 0 to 40) and divided into 3 categories (scores of 0–7; scores of 8–19; and scores > 19) [27]. The AUDIT is a screening tool that helps providers to assess patients’ alcohol related risks; a score of 7 and below suggests that person abstains or has lower-risk drinking. Individuals who score between 8 and 19 are at risk for consequences. A score of above 19 is suggestive of alcohol dependence [27, 28]. We defined an AUDIT score > 7 as unhealthy alcohol use.

We defined injection drug use as self-report of any past 30-day IDU (yes or no). Opioid craving was measured via a visual analogue scale ranging from 0 to 100, modeled using tertiles. The opioid craving measure was validated and used in prior studies [29, 30]. We did not model craving as a continuous variable in order to avoid assumptions of linearity.

#### Covariates

In the analysis of the primary outcome (linkage to care), the following potential confounders were included based on the literature and our clinical knowledge: age, gender, education, marital status, income, social support [31], depressive symptoms (Center for Epidemiologic Studies Depression Scale [CES-D]), [32, 33] homelessness, and HIV stigma (Berger HIV stigma scale) [34]. As LINC is an RCT, we also considered the study arm as a covariate.

### Statistical analyses

 Descriptive statistics were used to characterize study participants overall and stratified separately by each of the 3 main independent variables. For each of the 3 main independent variables, we presented baseline characteristics by each category of the particular substance use variable (e.g., as shown in Table 1, for the AUDIT score that measured alcohol use, baseline characteristics were presented for the following three categories: scores of 0–7; scores of 8–19; and scores > 19). We compared exposure groups for descriptive purposes using Chi square and Student’s t tests or Wilcoxon rank-sum tests, as appropriate. Spearman correlations were calculated to assess correlations between independent variables and covariates and no pair of variables included in the same regression model was highly correlated (r < 0.40 in all cases). Separate multiple logistic regression models were used to evaluate associations between each independent variable with each outcome adjusting for potential confounders. We reported adjusted odds ratios (aOR) and 95% confidence intervals (CI) from the regression models. For the secondary outcome, prescribed ART, due to a limited number of events (i.e., 31 prescribed ART within 12 months), we limited the adjusted analyses to the following covariates: age, gender, and stigma. As only 5 events for the undetectable viral load outcome were identified, we present only an unadjusted model for this outcome. Confirmatory analyses were conducted additionally adjusting for randomization to the LINC intervention in analyses of the primary outcome of linkage to HIV care and the secondary outcome of being prescribed ART within 12 months. We conducted analyses using 2-sided tests and an alpha level of 0.05. All statistical analyses were conducted using SAS version 9.3 (SAS Institute, Inc., NC, USA).

**Table 1:** Characteristics of HIV-positive Russians with opioid use, overall and by AUDIT score (n = 249)

Characteristic	Total N = 249	AUDIT^a^ score 0–7 n = 101	AUDIT^a^ score 8–19 n = 81	AUDIT^a^ score 20–40 n = 67	p value
Age: mean (SD)	34.3 (4.8)	34.6 (4.9)	34 (5.1)	34.2 (4.2)	0.70
Male	184 (74%)	72 (71%)	64 (79%)	48 (72%)	0.44
Married or partnered	84 (34%)	32 (32%)	30 (37%)	22 (33%)	0.52
Education (less than 9 grades)	67 (27%)	24 (24%)	24 (30%)	19 (28%)	0.36
Depressive symptoms CES-D ≥ 16	208 (88%)	79 (84%)	67 (88%)	62 (95%)	0.06
Social support: mean (SD)	19 (5)	19 (5)	19 (5)	19 (5)	0.85
Stigma score: mean (SD)^a^	2 (1)	2 (1)	2 (1)	2 (1)	0.62
Injection drug use, past-month^a^	130 (52%)	41 (41%)	51 (63%)	38 (57%)	< 0.001
Opioid craving: mean (SD)^a^	49 (38)	41 (37)	54 (36)	53 (41)	0.04
Linked to care	119 (48%)	48 (48%)	39 (48%)	32 (48%)	1.00
ART initiation	31 (12%)	13 (13%)	11 (14%)	7 (10%)	0.85

^a^Collected at 6 months from baseline

## Results

### Participant characteristics

Participants in the primary analysis of linkage to HIV care and the secondary analysis of prescription of ART (N = 249) are described in Tables 1 and 2. The subset of these participants with HIV viral load results (n = 48) were examined in the exploratory analysis of the cascade outcome, HVL suppression. Characteristics of this Russian HIV-positive cohort are the following: mean age 34 years (SD: 4.8); 74% men; 34% married, 24% separated and 42% never married; 27% completed 9 years or less of school, 62% completed 12 years of schooling, and 10% reported some higher education. Only 3% were homeless. Mean CD4 cell count at baseline was 365 cells/mm^3^ (SD: 260). The median monthly individual income of participants was 25,000 rubles (USD 775 [2013 exchange rate]). We used the median split approach [35] to dichotomize participants into 2 groups: lower than median income (0–25,000 rubles) or higher than median income (> 25,000 rubles). Of note, the minimum necessary income for an individual to meet basic needs (living wage) in St. Petersburg in 2013 was 6900 rubles (USD 214) [36]. Depressive symptoms were common, with 88% scoring above 16 on the CES-D [32].

**Table 2:** Characteristics of HIV-positive Russians with opioid use, overall and by past-month IDU status (n = 249)

Characteristic	Total N = 249	IDU past month^a^ n = 130	No IDU past month^a^ n = 119	p value
Age: mean (SD)	34.3 (4.8)	33.6 (5.1)	35.1 (4.3)	0.01
Male	184 (74%)	89 (69%)	95 (80%)	0.04
Married or partnered	83 (33%)	45 (35%)	38 (32%)	0.23
Education (less than 9 grades)	68 (27%)	39 (30%)	29 (24%)	0.06
Depressive symptoms CES-D ≥ 16	208 (88%)	113 (92%)	95 (85%)	0.10
Social support: mean (SD)	19 (5)	19 (5)	19 (5)	0.25
Stigma score: mean (SD)^a^	2 (1)	2 (1)	2 (1)	0.85
AUDIT^a^				
Score 0–7	100 (40%)	41 (31%)	59 (50%)	0.009
Score 8–19	81 (33%)	51 (39%)	30 (25%)	
Score 20–40	67 (27%)	38 (29%)	29 (25%)	
Opioid craving: mean (SD)^a^	49 (38)	71 (31)	24 (29)	< 0.001
Linked to care	119 (48%)	57 (43.8%)	62 (52%)	0.21
ART initiation	31 (12%)	15 (11%)	16 (13%)	0.70
CD4 cell count: mean (SD)	365 (260)	340 (256)	393 (264)	0.11

^a^Collected at 6 months from baseline

Unhealthy alcohol use was common, with a majority (59%) having an AUDIT score of 8 or higher. Past-month IDU was also common (52%). Unhealthy alcohol use occurred among 68% of those with past-month IDU (89/130). The mean opioid craving score was 49 (SD: 38). Variables indicative of the HIV care cascade were as follows: 119/249 participants (48%) were linked to HIV care; 31/249 (12%) were prescribed ART; 5/48 (10%) achieved viral suppression (HVL < 500 cells/mm^3^) within a year of study enrollment.

### Regression analyses

#### Linkage to HIV care

We were unable to detect significant associations between the linkage to care outcome and the independent variables (i.e., unhealthy alcohol use, IDU, and opioid craving) in unadjusted and adjusted analyses (Table 3). Adjusted odds ratio (aOR) for unhealthy alcohol use and linkage to care were as follows: 1.14 for AUDIT score of 20–40 (95% CI 0.57–2.29, p = 0.71) and 1.26 for AUDIT score of 8–19 (95% CI 0.65–2.24, p = 0.49) compared with people with lower-risk drinking and abstainers (AUDIT scores 0–7). Similarly, in both unadjusted and adjusted analyses, past-month IDU was not significantly associated with linkage to HIV care (aOR 0.79 [95% CI 0.45–1.38, p = 0.39]).

**Table 3:** Separate logistic regression models evaluating the association between substance use (unhealthy alcohol use, past-month IDU, opioid craving) and linkage to care (n = 249)

Variable	Outcome					
	Linkage to care and unhealthy alcohol use n = 249		Linkage to care and IDU n = 249		Linkage to care and opioid craving n = 250	
	Adjusted odds ratio (95% CI)	p value	Adjusted odds ratio (95% CI)	p value	Adjusted odds ratio (95% CI)	p value
AUDIT 20–40 Alcohol dependence	1.14 (0.57, 2.29)	0.72	–	–	–	–
AUDIT 8–19 Hazardous drinking	1.26 (0.65, 2.44)	0.49	–	–	–	–
IDU	–	–	0.79 (0.45, 1.38)	0.40	–	–
Opioid craving 30–70	–	–	–	–	0.78 (0.39, 1.57)	0.49
Opioid craving 71–100	–	–	–	–	0.84 (0.43, 1.64)	0.61
Gender (female vs. male)	1.45 (0.74, 2.84)	0.27	1.45 (0.74, 2.82)	0.27	1.39 (0.72, 2.71)	0.33
Age	1.00 (0.94, 1.07)	0.90	1.00 (0.93, 1.07)	0.93	1.00 (0.94, 1.07)	0.97
Stigma (continuous)	0.71 (0.44, 1.14)	0.16	0.71 (0.44, 1.14)	0.15	0.71 (0.44, 1.15)	0.16
Social support (continuous)	1.00 (0.94, 1.06)	0.95	1.00 (0.95, 1.07)	0.88	1.00 (0.94, 1.07)	0.91
Married or partnered	0.46 (0.24, 0.89)	0.02	0.45 (0.23, 0.87)	0.01	0.47 (0.24, 0.91)	0.02
Separated, divorced, or widowed	1.05 (0.49, 2.28)	0.89	1.09 (0.51, 2.32)	0.82	1.07 (0.50, 2.29)	0.85
Education	1.97 (1.02, 3.78)	0.04	1.91 (0.99, 3.68)	0.05	1.99 (1.03, 3.84)	0.04
Depressive symptoms (past-week symptoms)	0.83 (0.34, 2.00)	0.67	0.87 (0.36, 2.08)	0.74	0.90 (0.37, 2.19)	0.82
Income (high vs. low)	1.04 (0.55, 1.96)	0.91	1.09 (0.58, 2.02)	0.79	1.09 (0.58, 2.04)	0.78
Homeless	1.70 (0.30, 9.57)	0.54	1.75 (0.31, 9.93)	0.52	1.87 (0.33, 10.59)	0.48

We found no significant association between opioid craving and linkage to HIV care outcome in unadjusted or adjusted regression models (aOR 0.84, [95% CI 0.43–1.64, p = 0.61), highest (71–100) versus lowest (0–29) tertile; (aOR 0.78, [95% CI 0.39–1.57, p = 0.48]), middle (30–70) versus lowest tertile.

Married or partnered status was associated with significantly lower odds of linkage to care in alcohol use (0.46 [0.24, 0.89]), and other models, see Table 3. Stigma—another covariate in our analyses—was not significantly associated with HIV care cascade outcomes (p > 0.05 for all linkage to care models). However, more education, appeared to be positively associated with linkage to care in all models, for example aOR for education in the alcohol use and linkage to care model was 1.97 (95%CI 1.02, 3.78), p = 0.04. Our main findings were consistent after adjustment for randomization to the LINC intervention group (data not shown).

#### ART and suppressed HIV viral load

We did not find significant associations between the main independent variables (i.e., unhealthy alcohol use, IDU, and opioid craving) and secondary (prescription of ART) or exploratory (achievement of viral control) outcomes (Tables 4, 5). In fact, the estimated effects did not even suggest an association in the hypothesized direction that substance use factors examined were associated with worse HIV care cascade outcomes.

**Table 4:** Separate logistic regression models evaluating the association between substance use (unhealthy alcohol use, past-month IDU, opioid craving) and ART (n = 249)

Variable	Outcome					
	ART and unhealthy alcohol use n = 249 use		ART and IDU n = 249		ART and opioid craving n = 250	
	Adjusted odds ratio (95% CI)	p value	Adjusted odds ratio (95% CI)	p value	Adjusted odds ratio (95% CI)	p value
AUDIT 20-40 Alcohol dependence	0.98 (0.37, 2.57)	0.97	–	–	–	–
AUDIT 8–19 Hazardous drinking	1.24 (0.52, 2.95)	0.62	–	–	–	–
IDU	–	–	0.89 (0.41, 1.90)	0.76	–	–
Opioid Craving 30–70	–	–	–	–	1.34 (0.53, 3.37)	0.53
Opioid Craving 71–100	–	–	–	–	1.16 (0.46, 2.92)	0.76
Gender (female vs. male)	1.25 (0.52, 2.97)	0.61	1.23 (0.51, 2.93)	0.64	1.22 (0.51, 2.90)	0.65
Age	1.04 (0.97, 1.13)	0.28	1.04 (0.96, 1.13)	0.31	1.05 (0.97, 1.13)	0.27
Stigma	0.74 (0.39, 1.40)	0.35	0.73 (0.39, 1.40)	0.35	0.74 (0.39, 1.40)	0.35

**Table 5:** Separate logistic regression models evaluating associations between substance use (unhealthy alcohol use, past-month IDU, opioid craving) and HVL suppression (n = 49)

Variable	Outcome					
	Suppressed HVL and AUDIT		Suppressed HVL and IDU		Suppressed HVL and opioid craving	
	Odds ratio (95% CI)	p value	Odds ratio (95% CI)	p value	Odds ratio (95% CI)	p value
AUDIT 20–40 Alcohol dependence	3.07 (0.32, 29.06)	0.33	–	–	–	–
AUDIT 8–19 Hazardous drinking	1.77 (0.20, 15.82)	0.61	–	–	–	–
IDU	–	–	0.90 (0.15, 5.25)	0.90	–	–
Opioid craving 30–70	–	–	–	–	0.96 (0.16, 5.86)	0.97
Opioid craving 71–100	–	–	–	–	0.27 (0.01, 6.48)	0.42

## Discussion

### Substance use is not associated with the examined stages in the HIV care cascade in this cohort

Alcohol and drug use have been implicated in HIV disease transmission and progression, but the role of these behaviors in each step of the HIV care cascade is less explored, especially in Eastern Europe. In this cohort of HIV-positive Russians who have been in addiction hospital treatment in the past year and had a lifetime history of injection drug use, we did not find a major role of individual substance use characteristics in the HIV care cascade milestones. Given the high prevalence of substance use and HIV infection in Russia, examining such associations is important.

The impact of alcohol use on HIV outcomes has been examined in other settings, and while areas of uncertainty exist, collective evidence suggests that there are possible mechanisms by which alcohol may be related to HIV disease progression, via low medication adherence and suboptimal retention in care [13, 37–39]. Research suggests that heavy drinkers are less likely to receive a prescription for ART [40–42]. However, it is unknown which stages of the HIV care cascade are most affected by unhealthy alcohol use. Our analysis attempted to examine this question by looking at alcohol’s effect on different steps of the HIV care cascade. Similar to alcohol use, opioid use is a known barrier to HIV care [43]. Specific effects of opioids on HIV disease progression are not fully understood, although some insights have been gained [44–46]. For example, studies have demonstrated a negative effect on CD4 count with heroin withdrawal in Russia [44]. A recent cross-sectional study among PWID in St. Petersburg and Kohtla-Järve, Estonia demonstrated that high alcohol consumption and injection frequency are significantly associated with missing HIV care cascade steps [47].

### Systemic factors merit further investigation

In some countries, access to HIV care among people who inject drugs (PWID) is disproportionately low due to system level characteristics. Systemic factors such as provider discrimination and stigmatization of affected people, low quality of care, criminalization of drug use, or detention in camps without effective treatment [22, 48, 49] might play a more important role resulting in poor HIV cascade outcomes. An example of a system level barrier to HIV care is providers’ negative attitudes about PWID in France in the early 2000s, when people with active injection use were threefold more likely not to receive ART because physicians doubted their ability to adhere to the regimen [50]. In contrast, evidence suggests that systemic factors associated with successful HIV treatment outcomes include provision of quality alcohol and/or drug addiction treatment, having a regular source of primary care, and provider expertise with HIV care [11].

Contrary to our hypotheses, individual determinants of people’s substance use do not appear to be key factors driving HIV care in this study population of Russians discharged in the previous year from an addiction hospital. It is possible that in Russia, systemic factors (e.g., related to access to HIV treatment and receipt of quality services) were major determinants of the HIV care cascade.

### Infrastructural challenges

These findings from Russia suggest that individual substance use factors were not significantly associated with achieving HIV care cascade milestones. This was unexpected and raises the possibility that alternative systemic barriers may dominate over individual substance use specific issues. One such possibility is that the infrastructure for delivery of HIV care is inadequate. Although HIV clinics have in recent years been increasingly distributed across city neighborhoods, availability of HIV facilities may still have been limited at the time of the study, making accessing these sites difficult for those who do not live in close proximity. The relationship of such structural issues can be tested with access to appropriate geographical data and if demonstrated as a substantial burden to HIV care, could be addressed by further expansion of accessible facilities. However, at this time, this is a hypothesis that merits further investigation. There are also barriers to adequate addiction care for example opioid agonist therapy does not exist in Russia, and alcohol treatment guidelines are far from evidence-based. It is therefore challenging for providers to offer high-quality addiction treatment, which has been shown to improve HIV outcomes [11, 15, 24].

A substantial body of literature exists on the protective effects of education on HIV care; this seems to be the case in this cohort, as education was positively associated with achievement of HIV care cascade outcomes [51, 52]. Married or partnered status was associated with significantly lower odds of linkage to care, suggesting that participants who were single had more progress with this HIV cascade outcome. This finding is surprising, given that partnered status usually has beneficial effects on overall health outcomes and HIV care [53, 54]. It is possible that single participants in this Russian cohort lived with their parents, and were therefore more motivated and financially better positioned to receive HIV care. This hypothesis merits further investigation.

### Limitations

The results of this study should be interpreted with caution and several limitations should be considered. This is a secondary data analysis and there may be lack of power to detect the relationships of interest. Given that all participants in the study were hospitalized for a substance use disorder, one could posit that the association of substance use with HIV care cascade outcomes could have been significant if the sample included participants without a substance use disorder (i.e., abstainers) as a comparison group. Initiation of ART had a limited number of events which precluded analysis with regression models controlling for the full set of desired covariates. Also, for the same outcome, due to limited sample size, we did not conduct analyses restricting the sample to only those who were eligible for ART, based on the Russian Federation guidelines for the initiation of pharmacotherapy at the time of the study (i.e., CD4 < 350 cells/mm^3^) [55], but rather included all participants, regardless of their CD4 status. In addition, due to limited sample size, HVL suppression could not be examined in multivariate analyses.

## Conclusion

Unhealthy alcohol use, past-month injection drug use, and opioid craving do not appear to play a major role in achieving the HIV care cascade milestones (i.e., linkage to HIV care; prescribed ART; and achievement of suppressed viral load) among a cohort of HIV-positive Russians with history of IDU. Continuing to pursue an understanding of the systemic factors that contribute to successful HIV care cascade outcomes in populations of PWID will be key to meeting an ambitious United Nations’ goal of global elimination of HIV infection.

## Abbreviations

ART: antiretroviral therapy
PWID: people who inject drugs
UN: United Nations
IDU: injection drug use
LINC: Linking Infectious and Narcology Care study
RCT: randomized controlled trial
ARCH: Alcohol Research Collaboration on HIV/AIDS
HVL: HIV viral load
AUDIT: alcohol use disorder identification test
CES-D: Center for Epidemiologic Studies Depression Scale
AOR: adjusted odds ratio
CI: confidence intervals
SD: standard deviation
OAT: opioid agonist therapy
